# Clinicians’ attitudes and knowledge of medicinal cannabis in opioid dependence treatment clinics in New South Wales, Australia

**DOI:** 10.1186/s42238-025-00315-6

**Published:** 2025-08-16

**Authors:** Laila Parvaresh, Llewellyn Mills, Jaleh Gholami, Louisa Jansen, Nazila Jamshidi, Kate Baker, Christopher Tremonti, Marguerite Tracy, Adrian Dunlop, Nicholas Lintzeris

**Affiliations:** 1 South Eastern Sydney Local Health District Drug and Alcohol Services, 591 South Dowling Street, Surry Hills, New South Wales, 2010 Australia; 2 Discipline of Addiction Medicine, Central Clinical School, Faculty of Medicine and Health, The University of Sydney, New South Wales, Australia; 3 School of Population Health, University of New South Wales, New South Wales, Australia; 4 Drug and Alcohol Clinical Research and Improvement Network (DACRIN), New South Wales, Australia; 5 Edith Collins Centre (Translational Research in Alcohol Drugs and Toxicity), New South Wales, Australia; 6 Sydney Local Health District Drug and Alcohol Services, New South Wales, Australia; 7 CIARA State Wide Committee, NSW Ministry of Health, New South Wales, Australia; 8 New South Wales Poisons Information Centre, The Children’s Hospital Westmead, New South Wales, Australia; 9 Illawarra Shoalhaven Local Health District Drug and Alcohol Service, New South Wales, Australia; 10 St Vincents Health Network Alcohol & Drug Service, New South Wales, Australia; 11 Western Sydney Local Health District Drug Health Services, New South Wales, Australia; 12 General Practice Clinical School, Faculty of Medicine and Health, The University of Sydney, New South Wales, Australia; 13 Hunter New England Local Health District Drug and Alcohol Services, New South Wales, Australia; 14 School of Medicine and Public Health, College of Health, Medicine and Wellbeing, University of Newcastle, New South Wales, Australia; 15 Hunter Medical Research Program, Healthcare Transformation Research Program, New South Wales, Australia

**Keywords:** Medicinal cannabis, Clinicians, Attitudes, Knowledge, Confidence, Methadone, Buprenorphine

## Abstract

**Background:**

There are no prior studies investigating the attitudes and knowledge of opioid treatment program (OTP) clinicians on prescribed medicinal cannabis in OTP clients. This study examined the OTP clinicians' medicinal cannabis experience, knowledge, concerns, and educational needs.

**Methods:**

Staff from six public OTP services in New South Wales completed a medicinal cannabis survey. Staff included nurses, doctors, pharmacists, allied health, and consumer workers. Single-level regression models were used to estimate participants’ sex, role, and year of experience effect.

**Results:**

102 (63%) clinicians responded to the medicinal cannabis part of the survey, mostly female (n = 58, 56.9%), and more than half worked full-time (n = 54, 52.9%). Most of the participants (88.5%, 85/96) lacked experience providing medicinal cannabis, two in three (66.7%, 68/102) agreed to consider medicinal cannabis as a treatment for addressing cannabis use in OTP clients. Over 70% (71.5%, 73/102) expressed similar agreement to consider medicinal cannabis for other health conditions in OTP clients. More than half of the clinicians (54.2%, 52/96) expressed a lack of confidence in assisting clients with accessing medicinal cannabis, and were unfamiliar with current regulations (56.2%, 54/96). Clinicians expressed safety concerns regarding side effects such as driving-related problems (74%, 71/96), cognitive impairment (54.2%, 52/96), and cannabis dependence (54.2%, 52/96). The three conditions most endorsed as having sufficient evidence to support the use of tetrahydrocannabinol (THC)-based medicinal cannabis were palliative care symptom management (72.4%, 71/98), chronic pain (67.4%, 66/98), and multiple sclerosis (43.8%, 43/98). The three conditions most identified as having sufficient evidence to support the routine clinical use of cannabidiol (CBD)-based medicinal cannabis were chronic pain (64.9%, 63/97), palliative care (62.5%, 60/96), and sleep problems (44.8%, 43/96). The most common educational needs identified by participants were the evidence for the effectiveness of medicinal cannabis in cannabis dependence treatment (88.5%, 85/96), other health conditions (87.5%, 84/96), and indications and contraindications for using medicinal cannabis (87.5%, 84/96).

**Conclusion:**

Despite the interest in using medicinal cannabis for treating cannabis dependence and /or other health conditions, clinicians identified several barriers including limited experience, lack of confidence, and poor understanding of the regulatory framework.

**Supplementary Information:**

The online version contains supplementary material available at 10.1186/s42238-025-00315-6.

## Background

Cannabis is the most commonly used illicit drug used in Australia and around the world (Peacock et al. 2018). Cannabis use is associated with potential harms, including mental health (e.g., affective disorder, anxiety, psychosis, suicidal behaviour), dependence, physical health (e.g., respiratory and cardiovascular disease), and social harms (e.g., motor vehicle accidents, school dropouts, unemployment) (Organization 2016). Previous literature reports that cannabis is a common substance used by clients in opioid treatment programs (OTP) (e.g., methadone or buprenorphine/naloxone), and between 39 to 66% of people who are opioid dependent and participating in an OTP used cannabis in the past month (Budney et al. 1998; Bawor et al. 2015; Lake 2020; Centre for Alcohol and Other Drugs 2018; Balhara 2014; Reisfield et al. 2009). Many clients in OTP also have other comorbid conditions such as mental health issues (Santo et al. 2022a), chronic pain (Martell et al. 2007), and sleep problems (Santo et al. 2021). Opioid dependence is often associated with the presence of comorbid mental health disorders (Frei and Rehm 2002). According to a recent systematic review, the reported prevalence rates were 36% for depression, 29% for anxiety, and 18% for post-traumatic stress disorder (PTSD) (Santo et al. 2022b). This review also noted lifetime prevalence rates of 34% for antisocial personality disorder and 18.2% for borderline personality disorder (Santo et al. 2022b). Adverse childhood experiences (ACEs) are also known to be associated with earlier opioid initiation and higher opioid overdose rates (Stein et al. 2017), with one study reporting that around 80% of people treated for opioid dependence experienced at least one form of childhood trauma (Sansone et al. 2009). Sleep disorder (reported in 78–88% (Khazaie et al. 2016)), and chronic pain conditions (affecting 55–64% (Latif et al. 2021; Delorme et al. 2021; Hser et al. 2017)) are also highly prevalent comorbidities within people with opioid dependency.

The past decade has seen the emergence of medicinal cannabis programs in many countries. In Australia, mental health, pain, and sleep conditions are the most common reasons for seeking medicinal cannabis treatment (Lintzeris et al. 2016, 2022; MacPhail et al. 2022). This raises questions about the extent to which cannabis use by OTP clients is related to comorbid health conditions, and how this may impact approaches used to address cannabis use in OTP programs.

Community perspectives on prescribed medicinal cannabis use have changed considerably in recent years, particularly in Australia, following the legalisation of medicinal cannabis in 2016 (Lintzeris et al. 2016). Several studies have assessed health professionals’ perspectives, knowledge, and educational needs regarding medicinal cannabis internationally (Gardiner et al. 2019; Hordowicz et al. 2021; Weisman 2021; Wildberger 2019) and nationally (Bawa et al. 2022; Jacobs et al. 2019). Health professionals’ attitudes around prescribed medicinal cannabis are evolving, and there seems to be greater acceptance of utilising medicinal cannabis in various clinical settings despite a number of identified barriers (Gardiner et al. 2019; Hordowicz et al. 2021; Weisman 2021; Bawa et al. 2022; Rønne et al. 2021; Benson et al. 2020; Chandiok et al. 2021; Hewa-Gamage et al. 2019; Hallinan et al. 2021; Dobson et al. 2024). Previous systematic reviews of studies measuring health professionals’ perspectives and attitudes towards medicinal cannabis have highlighted that while health professionals are generally supportive of medicinal cannabis use in clinical practice (Gardiner et al. 2019; Hordowicz et al. 2021), they often lack sufficient knowledge regarding its effectiveness, prescribing rules, and regulations, and raise concerns regarding potential side effects (Gardiner et al. 2019; Hordowicz et al. 2021; Rønne et al. 2021), and associated harms (e.g., dependence, diversion, and nonmedical use) (Gardiner et al. 2019; Weisman 2021). Many health professionals have expressed the need for more education regarding medicinal cannabis (Hordowicz et al. 2021; Weisman 2021). Similar barriers have been identified in studies of Australian health care professionals: lack of knowledge regarding the pharmacology of medicinal cannabis, insufficient clinical evidence, safety concerns, and the medicolegal liability of prescribing unapproved medicines (Gardiner et al. 2019; Dobson et al. 2024; O’Rourke et al. 2022), navigating a complicated Australian regulatory authority (Therapeutic Goods Administration (TGA) systems), the high cost of medications for patients as medicinal cannabis is not subsidised, and clinician peers’ negative attitudes toward medicinal cannabis prescribers (Dobson et al. 2024), have all been identified as barriers to medicinal cannabis prescribing in Australia (Bawa et al. 2022; Chandiok et al. 2021; Hewa-Gamage et al. 2019; Hallinan et al. 2021; Dobson et al. 2024). One prior North American study of clinicians working in addiction treatment settings identified mixed attitudes toward medicinal cannabis, expressing concerns over the potential for abuse (64%) and an inadequate evidence base (59%); however, they also believed using medicinal cannabis is safe if utilised responsibly (70%) (Wildberger 2019).

Our previous study on OTP clinicians’ perspectives on their clients’ cannabis use (Parvaresh et al. 2024), clinicians identified problematic cannabis use in 56% of clients, 64% were willing to enhance their efforts to address cannabis use in their clinical practice, and 77% believed strategies to address cannabis use should be implemented within OTP services rather than referred to external providers. In addition to increased provision of counselling, withdrawal, and harm reduction services, 60% of OTP clinicians were keen on prioritising medicinal cannabis as one of the strategies to address cannabis use in OTP clients, despite most OTP clinicians (69%) reporting a lack of confidence in providing medicinal cannabis treatment (Parvaresh et al. 2024). Therefore, it is important to better understand clinicians’ attitudes, as well as the potential barriers and facilitators to using medicinal cannabis in OTP settings. To our knowledge, no prior study has investigated the attitudes, knowledge, concerns, and experience of clinicians working in opioid treatment settings regarding prescribed medicinal cannabis.

In this study, we aimed to explore OTP clinicians’ experience, knowledge, concerns, and educational needs related to medicinal cannabis in six different local health districts (LHDs) and health networks (HNs) in New South Wales (NSW), Australia.

## Methods

### Study design and setting

The data for this study were taken from a cross-sectional online survey investigating OTP clinicians’ attitudes and knowledge regarding cannabis use in OTP clients. OTP clinicians working in six LHDs and HNs OTP clinics in NSW, Australia participated in this study. Participating services are part of the NSW Drug and Alcohol Clinical Research and Improvement Network (DACRIN) (Centre for Alcohol and Other Drugs, Ministry of Health, NSW Health 2023), and comprise four metropolitan and two regional districts. The study was approved by the Sydney Local Health District Human Research Ethics Committee (X22-0327&2022/ETH01545).

### Participants and recruitment

Participants were staff employed in public-sector OTP services currently providing clinical care to outpatient OTP clients receiving methadone or buprenorphine. Participants’ professional backgrounds included nurses, medical practitioners, allied health workers, pharmacists, and consumer workers (see Table 1).

LHDs/HN site investigators emailed eligible staff weekly with invitations to participate in the study, providing links to the participant information sheet (PIS), electronic informed consent, and the online survey, for six weeks in March and April 2023. Participant data were collected anonymously. Site investigators estimated the total number of potentially eligible participants for each site, enabling an estimation of the participation rate.

### Study measures and data collection

The survey questionnaire was developed by the lead author in consultation with a project steering committee comprising clinicians and consumer worker representatives from the participating sites, was piloted by OTP clinicians for face validity, and subsequently refined. OTP clinicians who piloted the survey did not participate in the study. Medicinal cannabis questions were developed according to the study aims and were designed based on the previous studies on health professionals’ perspectives on medicinal cannabis (Weisman 2021; Jacobs et al. 2019) and modified for this study.

The “medicinal cannabis” section of the survey, contained 50 items across 5 sections:

1. OTP clinicians’ estimates of the percentage of the OTP clients who were accessing medicinal cannabis or had enquired about accessing prescribed medicinal cannabis (2 items, 7-point ordinal scale) and OTP clinicians’ level of agreement on whether medicinal cannabis should be available to clients to treat their cannabis use or other health conditions (2 items, 5-point Likert scale)
2. level of agreement on the strength of evidence for using THC/THC-CBD or CBD-based medicinal cannabis as first- or second-line treatment for various conditions (10 and 8 items, respectively, each on a 6-point Likert scale)
3. level of confidence in assisting OTP clients to access medicinal cannabis or understanding the regulatory requirements for its provision (2 items, 5-point Likert scale) and experience in providing medicinal cannabis to clients (one item)
4. level of concern about medicinal cannabis side effects (11 items, 5-point Likert scale)
5. The aspects of medicinal cannabis that participants would like to learn more about (10 items), and the preferred educational platforms (5 items)

The University of Sydney REDCap 13.4.10, a secure web platform for building and managing online surveys and databases, was used for data capture. A copy of the questionnaire is available in the Supplementary file (supplement 1).

### Statistical Analysis

Variables requiring respondents to estimate percentages of OTP clients accessing or enquiring about medicinal cannabis were presented as categorical variables with ranges of estimated percentages, 1–20%, 21–40%, 41–60%, 61–80%, and 81–100%. For purposes of analysis and ease of communicating the result (Guide n.d.), we converted these categorical variables to numerical variables by taking the mid-point of the category selected (e.g. if 61–80% was selected, it was assigned the number 70%).

Multiple regressions were used to analyse the data, one for each dependent variable. Regression models used for analysis were, a) linear regression model for continuous dependent variable (mid-point reported percentiles, e.g., percentage of total OTP clients that were accessing legally prescribed medicinal cannabis to treat any health condition), b) Bernoulli regression for binary (ever provided treatment with medicinal cannabis) or polytomous categorical variables (e.g., various preferred educational platforms), c) ordinal logistic regression model for ordinal categorical variables (e.g., various level of agreement on presence of evidence for effectiveness of CBD-based medicinal cannabis). The independent variables in all these models were role (four-level categorical: nurses [reference level] vs doctors vs allied health vs other), sex (male [reference] vs female), and years of experience (numeric, in years employed in OTP clinic: range [0–46]). As years of experience exhibited a significant correlation with age, verified by the Pearson test (correlation coefficient = 0.6, p-value < 0.001) and was more relevant to our study aims, years of experience was chosen.

A chi-square test was conducted to examine the correlation between “support for the provision of medicinal cannabis for the treatment of cannabis dependency or other health conditions” and “agreement on the presence of evidence for efficacy THC/THC-CBD or CBD-based medicinal cannabis in treating cannabis dependency or other health conditions”. Confidence intervals were adjusted for multiple comparisons utilising the Scheffe method (Scheffé 1953).

## Results

### Participant characteristics

Out of 162 clinicians working in public OTP services across the six LHDs/HN who consented to participate in a survey, 102 (63%) responded to the medicinal cannabis section of the survey and their data are reported here. The reasons for the non-completion of the survey were not collected.

The mean age of participants was 46.2 (± 13.2) years with a mean of 9.6 (± 9.9) years of experience working in OTP settings. Most participants were female (n = 58, 56.9%), and more than half worked full-time (n = 54, 52.9%) (see Table 1).

**Table 1:** Characteristics of clinicians

	**n (%)** **N = 102**
Sex	
Female	58 (56.9)
Male	40 (39.2)
Preferred not to answer	4 (3.9)
Age (years)	
Mean (SD)	46.2 (13.2)
Median (range; IQR)	46 (22–73; 34–58)
Employment locations	
Site 1	30 (29.4)
Site 2	21 (20.6)
Site 3	19 (18.6)
Site 4	15 (14.7)
Site 5	10 (9.8)
Site 6	7 (6.8)
Roles	
Nurses^1^	46 (45.1)
Doctors^2^	30 (29.4)
Allied health^3^	14 (13.7)
Others^4^	12 (11.8)
Employment hours per week	
Part-time: ≤ 20 h	34 (33.3)
Part-time: 21–30 h	14 (13.7)
Fulltime: ≥ 31 h	54 (52.9)
OTP employment history (years)	
Mean (SD)	9.6 (9.9)
Median (range; IQR)	5 (0–46; 2–15)

^1^Enrolled nurses, Registered Nurses, midwives, Clinical Nurse Specialists, Clinical Nurse Consultants, Nurse Unit Managers, Nurse practitioners, and Clinical Nurse Educators

^2^Junior Medical Officers (registrars or residents), Staff Specialists, Visiting Medical Officers, and Career Medical Officers

^3^Social workers, counsellors, psychologists

^4^Managers, pharmacists, and consumer workers

### Clinicians’ attitudes, confidence, and experience with the provision of medicinal cannabis

Most staff (88.5%, 85/96) lacked experience providing medicinal cannabis treatment. Few doctors (27.6%, 8/29) reported having experience of prescribing medicinal cannabis. More than two in three clinicians (66.7%, 68/102) agreed or strongly agreed to consider medicinal cannabis as a treatment option for addressing cannabis use in some OTP clients. Over 70% of clinicians (71.5%, 73/102) expressed similar agreement to consider medicinal cannabis for other health conditions in certain OTP clients. However, over half of the clinicians (54.2%, 52/96) reported a lack of confidence (“not at all confident” and “not very confident”) in assisting clients with accessing medicinal cannabis; and most were unfamiliar with current regulatory requirements for medicinal cannabis provision (56.2%, 54/96) (see Table 2). Overall, clinicians estimated that 13.8% (± 16.7) of their OTP clients were currently accessing legally prescribed medicinal cannabis to treat a clinical condition, and 18.1% (± 19.1) had enquired from their clinician about accessing medicinal cannabis products. There were no statistically significant differences based on participants’ sex, profession, years of experience, or multiple comparisons.

**Table 2:** Clinicians'perspective of using medicinal cannabis and level of confidence in assisting clients, understanding regulations

	Total N (%)
**Medicinal cannabis should be an option for some OTP clients to address:**	
**their cannabis use**	
Strongly disagree	6 (5.9)
Disagree	7 (6.9)
Unsure	21 (20.6)
Agree	46 (45.1)
Strongly Agree	22 (21.6)
**other health conditions**	
Strongly disagree	7 (6.9)
Disagree	1 (1.0)
Unsure	21 (20.6)
Agree	39 (38.2)
Strongly Agree	34 (33.3)
**Level of confidence for**	
**Assisting clients to access prescribed medicinal cannabis**	
Very confident	8 (8.3)
Somewhat confident	18 (18.7)
Unsure	18 (18.7)
Not very confident	36 (37.5)
Not at all confident	16 (16.7)
**Understanding the current regulatory requirement for medicinal cannabis provision in NSW**	
Very confident	8 (8.3)
Somewhat confident	18 (18.7)
Unsure	16 (16.7)
Not very confident	37 (38.5)
Not at all confident	17 (17.7)

### Staff concerns about side effects of medicinal cannabis

**Fig. 1:**
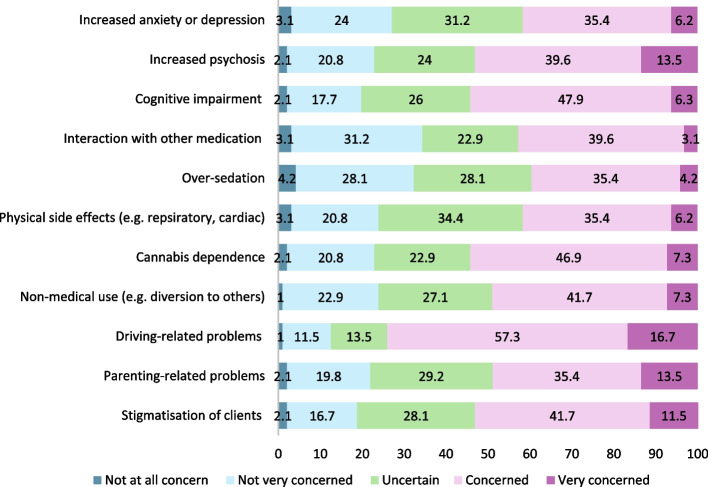
Perceived level of concerns for various side effects of medicinal cannabis by clinicians in percentage

Most clinicians reported being “concerned or very concerned” about driving-related problems (74%, 71/96), cognitive impairment (e.g., memory, slowed thinking) (54.2%, 52/96), cannabis dependence (54.2%, 52/96), increased psychosis and stigmatisation (53.1%, 51/96, each). They also reported being “less concerned or not concerned at all” about medicinal cannabis interactions with other medications (34.4%, 33/96), oversedation (32.3%, 31/96), and increased anxiety and depression (27.1%, 26/96). There were no statistically significant differences in the level of concern between different professions, adjusted for sex, and years of experience. However, each additional year of employment in alcohol and other drug services was associated with a 5% decrease in concern for increased anxiety or depression (adjusted Odds Ratio [aOR]: 0.95; CI: 0.91, 0.99), a 7% decrease for increased psychosis (aOR: 0.93; CI: 0.89, 0.97), a 5% decrease for parenting related problems (aOR: 0.95; CI: 0.91, 0.99), and 4% decrease for driving-related problems (aOR; 0.96; CI:0.92, 0.99). Female clinicians were more concerned about medicinal cannabis interacting with other medications (aOR:2.7; CI: 1.1, 6.7) and parenting-related problems (aOR: 3.1; CI: 1.3, 7.7) after adjusting for years of experience, profession, and multiple comparisons (Fig. 1).

### Staff agreement about sufficiency of evidence for using THC/THC-CBD or CBD-based medicinal cannabis as first- or second-line treatment for various conditions

#### THC/THC-CBD based medicinal cannabis

**Fig. 2:**
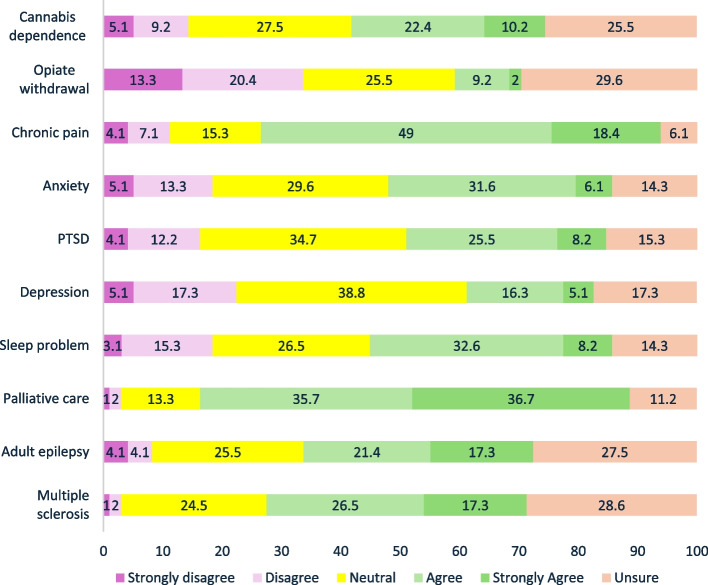
OTP clinicians’ beliefs for the presence of sufficient evidence to provide THC/THC-CBD medicine

The three conditions most identified by clinicians as having sufficient evidence to support the use of THC or THC-CBD medicinal cannabis as a first- or second-line treatment were palliative care symptom management (72.4%, 71/98), chronic pain (67.4%, 66/98), and multiple sclerosis (43.8%, 43/98) (see Fig. 2). The conditions for which clinicians had the most disagreement that, ‘there was sufficient evidence’, were opiate withdrawal (33.7%, 33/98 disagreed), depression (22.4%, 22/98 disagreed), anxiety (18.4%,18/98 disagreed), and sleep problems (18.4%, 18/98 disagreed). The conditions that clinicians most indicated that ‘evidence for efficacy was not clear or equivocal’ – indicated by the proportion of ‘Neutral’ responses—were depression (38.8%, 38/98), Post Traumatic Stress Disorder (PTSD) (34.7%, 34/98), and anxiety (29.6%, 29/98). The condition that clinicians were most commonly ‘unsure about, the presence of any evidence for THC/THC-CBD medicinal cannabis efficacy’, was the treatment of opiate withdrawal (29.6%, 29/98). Approximately a third (32.6%) of clinicians agreed that there was sufficient evidence for THC-based medicines to treat cannabis dependence, with only 14.3% disagreeing and 27.5% unsure.

Doctors were less likely than nurses to agree on there was an adequate level of evidence for the use of THC based medicines for the treatment of opiate withdrawal (aOR = 0.12; CI = 0.02, 0.79), chronic pain (aOR: 0.10; CI: 0.01, 0.54), anxiety (aOR: 0.13; CI: 0.02, 0.73), PTSD (aOR:0.14; CI: 0.02, 0.84), depression (aOR:0.14; CI:0.02, 0.84), and sleep problems (aOR:0.10; CI: 0.01, 0.60) after adjusting for sex, years of experience, and multiple comparisons.

#### CBD-based medicinal cannabis

**Fig. 3:**
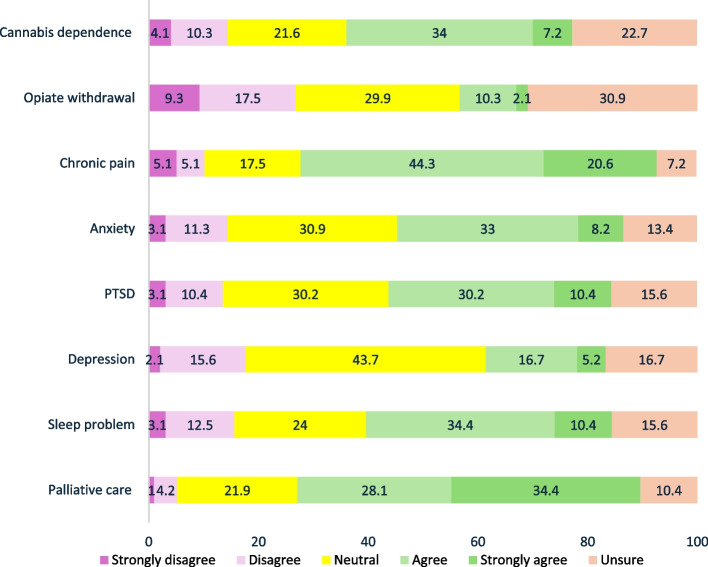
OTP clinicians’ beliefs about the presence of sufficient evidence to provide CBD-based medicine

The three conditions most identified by clinicians as having sufficient evidence to support the routine clinical use of CBD-based medicinal cannabis as first-line or second-line treatment were chronic pain (64.9%, 63/97), palliative care (62.5%, 60/96), and sleep problems (44.8%, 43/96). The conditions in which clinicians expressed the most disagreement that ‘there is sufficient evidence’ were opiate withdrawal (26.8%, 26/97 disagreed), depression (17.7%, 17/96 disagreed), and sleep problems (15.6%, 15/96 disagreed). The conditions that clinicians most stated that ‘evidence for efficacy for CBD was not clear or equivocal’- indicated by the proportion of ‘Neutral’ responses- were depression (43.7%, 42/96), anxiety (30.9%, 30/97), and PTSD (30.2%, 29/96). The conditions that clinicians were most commonly ‘unsure about the presence of any evidence for CBD-based medicinal cannabis efficacy’ were the treatment of opiate withdrawal (30.9%, 30/97) (see Fig. 3). Compared to nurses, doctors expressed significantly less agreement about the adequacy of evidence for using CBD-based medicinal cannabis as a first- or second-line treatment in the following conditions; chronic pain (aOR: 0.15; CI:0.03, 0.76), PTSD (aOR: 0.14; CI: 0.02, 0.87), and sleep problems (aOR: 0.12; CI:0.02, 0.77) adjusted for sex, years of experience and multiple comparisons.

All in all, among clinicians who supported the provision of medicinal cannabis for the treatment of cannabis dependence (68.7%, 55/80), a significantly higher proportion believed there is evidence supporting its efficacy for the treatment of cannabis dependency compared to those who thought there is no such evidence (80.8%, 38/47 versus 51.5%, 17/33, p-value = 0.005).

### Education and training needs

Clinicians expressed interest in gaining knowledge about various aspects of medicinal cannabis (see Table 3). The most common educational needs identified by participants were the evidence for effectiveness of medicinal cannabis in the treatment of cannabis dependency (88.5%, 85/96), the evidence of effectiveness for other health conditions (87.5%, 84/96), indications and contraindications for using medicinal cannabis (87.5%, 84/96), side effects (84.4, 81/96) and regulatory frameworks and procedures (84.4, 81/96). Female clinicians were significantly more interested in learning about ‘regulatory framework(s) for accessing medicinal cannabis’ and ‘cost of medicinal cannabis’, adjusting for years of experience and profession (aOR: 4.5; CI: 1.1, 18.4, aOR: 7.4; CI: 2.1, 25.9 respectively). Clinicians identified a need for clinical practice guidelines (86.5%, 83/96), and further education indicated the training to be delivered through in-person or webinar training sessions (88.5%, 85/96) (see Table 3). There were no statistically significant differences between different professional categories adjusted for years of experience and multiple comparisons.

**Table 3:** OTP clinicians’ education needs and educational platforms

	Total N (%)
**Aspects of medicinal cannabis that clinical staff would like to know more about**	
Evidence of efficacy for treating cannabis dependence	85 (88.5)
Evidence of efficacy in treating other health conditions	84 (87.5)
Indications and contraindications for medicinal cannabis	84 (87.5)
Adverse events and their management	81 (84.4)
Interaction with other medications and other substances	75 (78.1)
Types of medicinal cannabis preparations	77 (80.2)
Regulatory framework for accessing medicinal cannabis	81 (84.4)
Pharmacokinetics	69 (71.9)
Cost of medicinal cannabis products	72 (75.0)
Other	5 (5.2)
**Preferred education platform**	
Clinical practice guidelines	83 (86.5)
Training programs (face-to-face or online webinars)	85 (88.5)
Self-directed E-learning	59 (61.5)
Peer-reviewed literature (e.g., journal papers)	49 (51.0)

## Discussion

This study demonstrated that more than 60% of survey respondents agreed or strongly agreed to consider medicinal cannabis as a treatment option for addressing cannabis use or some other health conditions in certain OTP clients and identified potential barriers and facilitators to the implementation of medicinal cannabis treatment in OTP settings. The barriers included a lack of experience among most OTP clinicians in providing medicinal cannabis treatment, low levels of confidence in assisting OTP clients with accessing medicinal cannabis, limited understanding of the regulations around medicinal cannabis provision, and a lack of training and clinical guidelines. Clinicians expressed concerns about the side effects of medicinal cannabis, and they were less concerned about some side effects such as anxiety, depression, increased risk of psychosis, and parenting-related problems, with increased years of employment. In contrast, female clinicians were more concerned about parenting-related issues and interaction with other medications in comparison with male clinicians. There was considerable variation in the clinician’s appraisal of the available evidence for the effectiveness of THC or CBD-based medications in treating different medical conditions.

Nevertheless, most staff were open to considering medicinal cannabis treatment in this client population and demonstrated a strong interest in further education on this topic. These findings build on our previous study on clinicians’ perspectives about cannabis use in OTP clients attending NSW public outpatient OTP services (Parvaresh et al. 2024). In that study, clinicians identified cannabis use to be a problem in 41% of their clients. They prioritised using medicinal cannabis as one of the preferred strategies (alongside withdrawal services, harm reduction interventions, and counselling) to address cannabis use (Parvaresh et al. 2024).

Most of the staff (89%) lacked experience in providing medicinal cannabis treatment, and few doctors (27%) had experience in providing medicinal cannabis for their clients. This reflects that at the time of writing, a NSW Health Policy Directive restricts the use of medicines unapproved by the Australian regulatory authority (TGA) (NSW Health 2022) such that clinicians working in public sector services cannot prescribe medicinal cannabis to patients without attending to considerable administrative and regulatory hurdles. This compounds other barriers to providing medicinal cannabis treatment identified by our participants. Whilst few clinicians reported being confident in providing medicinal cannabis treatment, they nevertheless identified considerable consumer demand from their clients, estimating that 13% of their clients were using medicinal cannabis from other healthcare providers, and almost one in five clients (18%) had asked about accessing medicinal treatment. This gap between clinician confidence and consumer demand highlights the need for OTP clinicians to be better equipped in discussing and potentially providing medicinal cannabis treatment options for their clients, in addition to addressing the regulatory barriers that restrict doctors in public service from prescribing medicinal cannabis (NSW Health 2022).

As in prior studies of health care professionals, OTP clinicians in our study expressed safety concerns regarding side effects such as driving-related problems, cognition, and cannabis dependence. A systematic review of studies among health professionals found a similar proportion of clinicians raising concerns about patients developing a dependency on prescribed medicinal cannabis (57.8% versus 54.2%) (Weisman 2021). The proportion of OTP clinicians expressing concerns about cognitive impairment (e.g., memory and slowed thinking) was similar to a previous study conducted among neurologists about cannabis effects on Parkinson's disease (54% versus 55%) (Bega et al. 2017). Our OTP clinicians’ level of concern about drug-drug interactions was similar to the reported concern in other studies (32.3% versus 38.7% and 32%) (Carlini et al. 2017; Philpot and Ebbert 2019), although a lower proportion of OTP clinicians were concerned about driving-related harm compared to a study of neurologists (74% versus 96%) (Bega et al. 2017). In summary, in comparison to other studies, a similar proportion of OTP clinicians expressed safety concerns regarding medicinal cannabis as clinicians working in other health sectors.

We identified considerable variability among clinicians regarding their beliefs concerning the presence of evidence for the safety and efficacy of THC and CBD-based medicines to treat various conditions. Whilst most clinicians identified that there is sufficient evidence to support the use of CBD or THC-based medicines for chronic pain and palliative care, for all other conditions, there was considerable diversity between clinicians regarding whether the evidence supported the use of medicinal cannabis for different conditions, suggesting little consensus between staff. For example, 33% and 41% of respondents believed there was sufficient evidence to support the use of THC-based medicine and CBD for treating cannabis dependence, 14% felt the evidence was insufficient for both, 28% and 22% found the evidence equivocal for THC-based medicine and CBD, respectively. In comparison, 26% and 22% were uncertain about their effectiveness. Regarding opioid dependence treatment – few clinicians identified there to be sufficient evidence to promote the use of THC-based medicine (11%) or CBD (12%) to assist in the management of opioid withdrawal, some respondents identifying the evidence did not support the use of THC-based medicine (34%) nor CBD (27%) for this indication, some were equivocal for THC-based medicine (25%) or CBD (30%) and uncertain for THC-based medicine (30%) or CBD (31%). Previous systematic reviews also demonstrated mixed results for the efficacy of CBD or THC-based medicine for the treatment of cannabis dependency (Bahji and Mazhar 2016; Nielsen et al. 2019), and opiate withdrawal (Babalonis and Walsh 2020). Regarding highly prevalent conditions such as depression, anxiety, and PTSD in people with opioid dependence, some clinicians felt there was sufficient evidence for effectiveness of THC-based medicine (38%) or CBD (41%) for treatment of anxiety, some were equivocal for THC-based medicine (27%) or CBD (31%), and few did not agree with presence of sufficient evidence for treatment of anxiety by THC-based medicine (18%) or CBD (14%). This is consistent with studies showing mixed results for the efficacy of medicinal cannabis in treating anxiety, some demonstrating some anxiolytic effects for CBD (Berger et al. 2022), some mixed effects for THC-based medicinal cannabis (Stack et al. 2022) and clinical trials reporting equivocal findings, contrasting with reported anxiolytic effects in large-scale surveys (Ameringen et al. 2020). Clinicians reported a similar level of agreement for the effectiveness of medicinal cannabis in treating PTSD. Some identified sufficient evidence for THC-based medicinal cannabis (34%) or CBD (41%). Others were equivocal about THC-based (35%) or CBD (30%). Few believed there was insufficient evidence for THC-based (16%) or CBD (13%). For depression, some clinicians were neutral for the presence of evidence for THC-based medicinal cannabis (39%) or CBD (44%), and fewer clinicians agreed with the presence of evidence for THC-based medicinal cannabis (21%) or CBD (22%) and others believed there is insufficient evidence for either THC-based medicinal cannabis (22%) or CBD (18%). This divergence in level of agreement for both PTSD and depression may be driven by the lack of large-scale randomised controlled trials to make a definitive conclusion about the efficacy of medicinal cannabis for treating PTSD and depression, despite some promising preclinical evidence (Orsolini et al. 2019; Yarnell 2015; Martin et al. 2021; Pavanetti et al. 2024).

A recent Australian study identified divergent perspectives regarding the evidence base for medicinal cannabis as a key barrier for clinicians considering prescribing medicinal cannabis (Benson et al. 2020). This diversity in clinicians’ perspectives highlights the need for a comprehensive and systematic collation of the available evidence and clinical guidance documents (NSW Health 2023) and their synthesis into clinical recommendations in up-to-date, evidence-based guidelines. In our survey, 87% of respondents identified the need for clinical guidelines regarding medicinal cannabis.

Most OTP clinicians expressed willingness to consider medicinal cannabis for treating various health conditions or cannabis use disorder in some OTP clients. This finding aligns with another study conducted among American addiction clinicians, demonstrating nearly two-thirds of addiction clinicians were in favour of using medicinal cannabis for clients undergoing substance use treatment (Wildberger 2019).

Similar to previous studies of health professionals (Gardiner et al. 2019), the majority of OTP clinicians in our survey expressed interest in improving their knowledge regarding clinical aspects of medicinal cannabis (e.g. pharmacology, dosing guides, side effects, drug-drug interactions), logistics of treatment (e.g. cost of products, regulatory frameworks), the evidence regarding the efficacy of medicinal cannabis in treating cannabis dependency and other health conditions, and indications or contraindications of using medicinal cannabis. Clinicians predominantly preferred receiving education through face-to-face workshops online webinars, training programs, and clinical practice guidelines, rather than self-directed learning or journal papers, mirroring findings from earlier studies (Bega et al. 2017).

## Limitations

The study relied on convenience sampling – and whilst a high proportion of eligible staff participated in the survey (approximately one-third of the eligible 300 clinicians working in OTP across the participating services), the study may have recruited staff with particular perspectives regarding medicinal cannabis (either for or against), therefore, there is a possibility of selection bias. The survey was done only in outpatient public settings by OTP clinicians and may not reflect the perspectives of clinicians working in private healthcare settings.

## Conclusion

Over two-thirds of survey respondents working in opioid treatment programs, considered medicinal cannabis to be an appropriate treatment modality to treat cannabis dependence and/or other conditions that commonly occur in this patient population, such as chronic pain and mental health conditions. However, several barriers to the use of medicinal cannabis in opioid treatment settings were identified, including limited experience and confidence of staff in providing medicinal cannabis treatment and a poor understanding of regulatory frameworks by most clinicians. Clinicians expressed concerns about the side effects of medicinal cannabis, such as impaired cognition, driving, and the potential for cannabis dependence, and there was considerable variation in their appraisal of the available evidence regarding the efficacy of THC or CBD-based medications in treating different medical conditions. Clinicians were keen to participate in training programs and sought evidence-based clinical guidelines to address some of these barriers. These findings highlight the need for robust systematic reviews on the effectiveness of various formulations of medicinal cannabis, the development of tailored educational programs for public OTP clinicians, and the establishment of national clinical guidelines on medicinal cannabis to support medicinal cannabis provision in public OTP clinics.

## Supplementary Information

Supplementary Material 1.

## Data Availability

No datasets were generated or analysed during the current study.

## Abbreviations

OTP: Opioid therapeutic program
DACRIN: Drug and Alcohol Clinical Research and Improvement Network
THC: Tetrahydrocannabinol
CBD: Cannabidiol
LHD: Local health district
HNs: Health networks
PIS: Participant information sheet
PTSD: Post Traumatic Stress Disorder
aOR: Adjusted Odds Ratio
TGA: Therapeutic Goods Administration
