# Supplementary material for: Clinicians’ attitudes and knowledge of medicinal cannabis in opioid dependence treatment clinics in New South Wales, Australia
Source: J Cannabis Res. 2025 Aug 16;7:59. doi: 10.1186/s42238-025-00315-6 (PMC12357479; doi:10.1186/s42238-025-00315-6)
Supplement: Supplementary file 1 — Supplementary Material 1. [file 42238_2025_315_MOESM1_ESM.pdf]

Clinicians' perspectives on medicinal cannabis in opioid dependence treatment clinics in New South Wales, Australia

Sociodemographic		
1.1	How would you identify yourself?	o Man, or male<br>o Woman, or female<br>o I use a different term<br>o Prefer not to answer
1.1a	Please tell us which gender identity you most readily identify as.	-----------------------------------<br>(Please specify)
1.2	Please let us know how old you are in years.	-----------------------------------<br>(Years old)
1.3	Which Local Health District or Health Network are you employed at?	
1.4	In what capacity are you employed at your Local Health District or Local Health Network?	o Doctor<br>o Registered Nurse<br>o Psychologist<br>o Social worker<br>o Consumer worker<br>o Pharmacist<br>o Manager<br>o Other<br>------------------------------------<br>(Please specify)
1.4a	Please tell us in what level you are practicing? (if "Doctor")	o Staff specialist<br>o Registrar<br>o Resident Medical Officer<br>o Career Medical Officer<br>o Visiting Medical Officer
1.4b	Please tell us at what level of nursing you are practicing? (if "Registered Nurse")	o RN 1-3<br>o RN 4-8<br>o CNS1 or 2<br>o CNC<br>o NUM or NM
1.5	How many years have you worked in Opioid Treatment Program (OTP) services? (please provide a number).	---------------------------- (yrs)
1.6	How many hours do you currently spend on average working in OTP each week?	o Less than 10 hours<br>o 11-20 hours<br>o 21-20 hours<br>o 31-40 hours<br>o More than 40 hours
1.7	What is your average OTP case load in the last three months (number of clients for whom you prescribe, or case manage or provide clinical care)	--------------------------------------<br>(Please provide a number)

**Medicinal Cannabis**		
The following questions concern your perspectives on medicinal cannabis (e.g., THC, CBD, and THC-CBD based medicines)		
5.1	I believe that medicinal cannabis should be an option for some OTP clients to address their cannabis use.	○ Strongly disagree<br>○ Disagree<br>○ Unsure<br>○ Neutral<br>○ Agree<br>○ strongly agree
5.2	I believe that medicinal cannabis should be an option for some OTP clients to address other health conditions (e.g., pain, mental health, sleep, palliative care).	○ Strongly disagree<br>○ Disagree<br>○ Unsure<br>○ Neutral<br>○ Agree<br>○ strongly agree
5.3	What percentage of *your total OTP client caseload* do you think are currently accessing legally prescribed medicinal cannabis to treat any clinical condition?	○ 0<br>○ 1-20%<br>○ 21-40%<br>○ 41-60%<br>○ 61-80%<br>○ 81-100%<br>○ Do not know
5.4	What percentage of *your total OTP client caseload* have asked you about accessing medicinal cannabis products?	○ 0<br>○ 1-20%<br>○ 21-40%<br>○ 41-60%<br>○ 61-80%<br>○ 81-100%<br>○ Do not know
5.5	Please indicate the extent to which you "agree or disagree" with each of the following statements:<br><br>"There is strong evidence for the use of medicinal cannabis containing **THC or THC-CBD combination** to treat the following conditions."<br><br>Neutral: The evidence is NOT CLEAR/EQUIVOCAL.<br><br>Unsure: I am NOT FAMILIAR with evidence.<br><br>Disagree: There IS NO STRONG evidence (insufficient to support routine clinical use as first- or second-line treatment).<br><br>Agree: There IS STRONG evidence (sufficient to support routine clinical use as first- or second-line treatment).	
	a. Cannabis dependence	○ Strongly disagree<br>○ Disagree<br>○ Neutral<br>○ Agree<br>○ strongly agree<br>○ Unsure

	b. Opiate withdrawal	○ Strongly disagree<br>○ Disagree<br>○ Neutral<br>○ Agree<br>○ strongly agree<br>○ Unsure
	c. Chronic pain	○ Strongly disagree<br>○ Disagree<br>○ Neutral<br>○ Agree<br>○ strongly agree<br>○ Unsure
	d. Anxiety	○ Strongly disagree<br>○ Disagree<br>○ Neutral<br>○ Agree<br>○ strongly agree<br>○ Unsure
	e. PTSD	○ Strongly disagree<br>○ Disagree<br>○ Neutral<br>○ Agree<br>○ strongly agree<br>○ Unsure
	f. Depression	○ Strongly disagree<br>○ Disagree<br>○ Neutral<br>○ Agree<br>○ strongly agree<br>○ Unsure
	g. Sleep problems	○ Strongly disagree<br>○ Disagree<br>○ Neutral<br>○ Agree<br>○ strongly agree<br>○ Unsure
	h. Palliative care	○ Strongly disagree<br>○ Disagree<br>○ Neutral<br>○ Agree<br>○ strongly agree<br>○ Unsure
	i. Epilepsy	○ Strongly disagree<br>○ Disagree<br>○ Neutral<br>○ Agree<br>○ strongly agree<br>○ Unsure
	j. Multiple sclerosis	○ Strongly disagree<br>○ Disagree

		○ Neutral<br>○ Agree<br>○ strongly agree<br>○ Unsure
5.6	Please indicate the extent to which you "agree or disagree" with each of the following statements:<br><br>"There is strong evidence for the use of **CBD-based medicinal cannabis** to treat the following conditions."<br><br>Neutral: The evidence is NOT CLEAR/EQUIVOCAL.<br><br>Unsure: I am NOT FAMILIAR with evidence.<br><br>Disagree: There IS NO STRONG evidence (insufficient to support routine clinical use as first- or second-line treatment).<br><br>Agree: There IS STRONG evidence (sufficient to support routine clinical use as first- or second-line treatment).	
	a. Cannabis dependence	○ Strongly disagree<br>○ Disagree<br>○ Neutral<br>○ Agree<br>○ strongly agree<br>○ Unsure
	b. Opiate withdrawal	○ Strongly disagree<br>○ Disagree<br>○ Neutral<br>○ Agree<br>○ strongly agree<br>○ Unsure
	c. Chronic pain	○ Strongly disagree<br>○ Disagree<br>○ Neutral<br>○ Agree<br>○ strongly agree<br>○ Unsure
	d. Anxiety	○ Strongly disagree<br>○ Disagree<br>○ Neutral<br>○ Agree<br>○ strongly agree<br>○ Unsure
	e. PTSD	○ Strongly disagree<br>○ Disagree<br>○ Neutral<br>○ Agree<br>○ strongly agree<br>○ Unsure

	f. Depression	○ Strongly disagree<br>○ Disagree<br>○ Neutral<br>○ Agree<br>○ strongly agree<br>○ Unsure
	g. Sleep problems	○ Strongly disagree<br>○ Disagree<br>○ Neutral<br>○ Agree<br>○ strongly agree<br>○ Unsure
	h. Palliative care	○ Strongly disagree<br>○ Disagree<br>○ Neutral<br>○ Agree<br>○ strongly agree<br>○ Unsure
5.7	How confident are you in assisting clients to access prescribed medicinal cannabis?	○ Very confident<br>○ Somewhat confident<br>○ Unsure<br>○ Not very confident<br>○ Not at all confident
5.8	How confident are you regarding your understanding of the current regulatory requirements to provide medicinal cannabis to a client in NSW?	○ Very confident<br>○ Somewhat confident<br>○ Neutral<br>○ Not very confident<br>○ Not at all confident
5.9	Have you ever provided treatment with medicinal cannabis products for any client (not just OTP clients)	○ Yes<br>○ No
5.10	How concerned are you about each of the following potential adverse events / side effects of medicinal cannabis?	
	a. Increased anxiety or depression	○ Not at all concerned<br>○ Not very concerned<br>○ Uncertain<br>○ Concerned<br>○ Very concerned
	b. Increased psychosis	○ Not at all concerned<br>○ Not very concerned<br>○ Uncertain<br>○ Concerned<br>○ Very concerned
	c. Cognitive impairment (e.g. memory, slowed thinking)	○ Not at all concerned<br>○ Not very concerned<br>○ Uncertain<br>○ Concerned<br>○ Very concerned

	d. Interaction with other medications	○ Not at all concerned<br>○ Not very concerned<br>○ Uncertain<br>○ Concerned<br>○ Very concerned
	e. Over-sedation	○ Not at all concerned<br>○ Not very concerned<br>○ Uncertain<br>○ Concerned<br>○ Very concerned
	f. Physical side effects (e.g. respiratory, cardiac)	○ Not at all concerned<br>○ Not very concerned<br>○ Uncertain<br>○ Concerned<br>○ Very concerned
	g. Cannabis dependence	○ Not at all concerned<br>○ Not very concerned<br>○ Uncertain<br>○ Concerned<br>○ Very concerned
	h. Non-medical use (e.g. diversion to others)	○ Not at all concerned<br>○ Not very concerned<br>○ Uncertain<br>○ Concerned<br>○ Very concerned
	i. Driving-related problems	○ Not at all concerned<br>○ Not very concerned<br>○ Uncertain<br>○ Concerned<br>○ Very concerned
	j. Parenting-related problems	○ Not at all concerned<br>○ Not very concerned<br>○ Uncertain<br>○ Concerned<br>○ Very concerned
	k. Stigmatisation of clients	○ Not at all concerned<br>○ Not very concerned<br>○ Uncertain<br>○ Concerned<br>○ Very concerned
	l. Others (please list)	--
5.11	What aspects of treatment with medicinal cannabis would you like to know more about (tick all that apply)	○ Evidence of efficacy of medicinal cannabis for treating cannabis dependence<br>○ Evidence of efficacy of medicinal cannabis in treating other health conditions

		○ Indications and contraindications for medicinal cannabis<br>○ Adverse events and their management<br>○ Interaction with other medications and other substances<br>○ Types of medicinal cannabis preparations<br>○ Regulatory framework for accessing medicinal cannabis<br>○ Pharmacokinetics<br>○ Cost of medicinal cannabis products<br>○ Other
5.11.a	If "other", please specify.	------------------
5.12	Which education platform would be useful in providing education for prescribing medicinal cannabis? (tick all that applies)	○ Clinical practice guidelines<br>○ Training programs (face-to-face or online webinars)<br>○ Self-directed E-learning<br>○ Peer-reviewed literature (e.g., journal papers)<br>○ Other
